# Supplementary figures and images for: Single cell transcriptional analysis reveals novel innate immune cell types
Source: PeerJ. 2014 Jun 24;2:e452. doi: 10.7717/peerj.452 (PMC4081288; doi:10.7717/peerj.452)

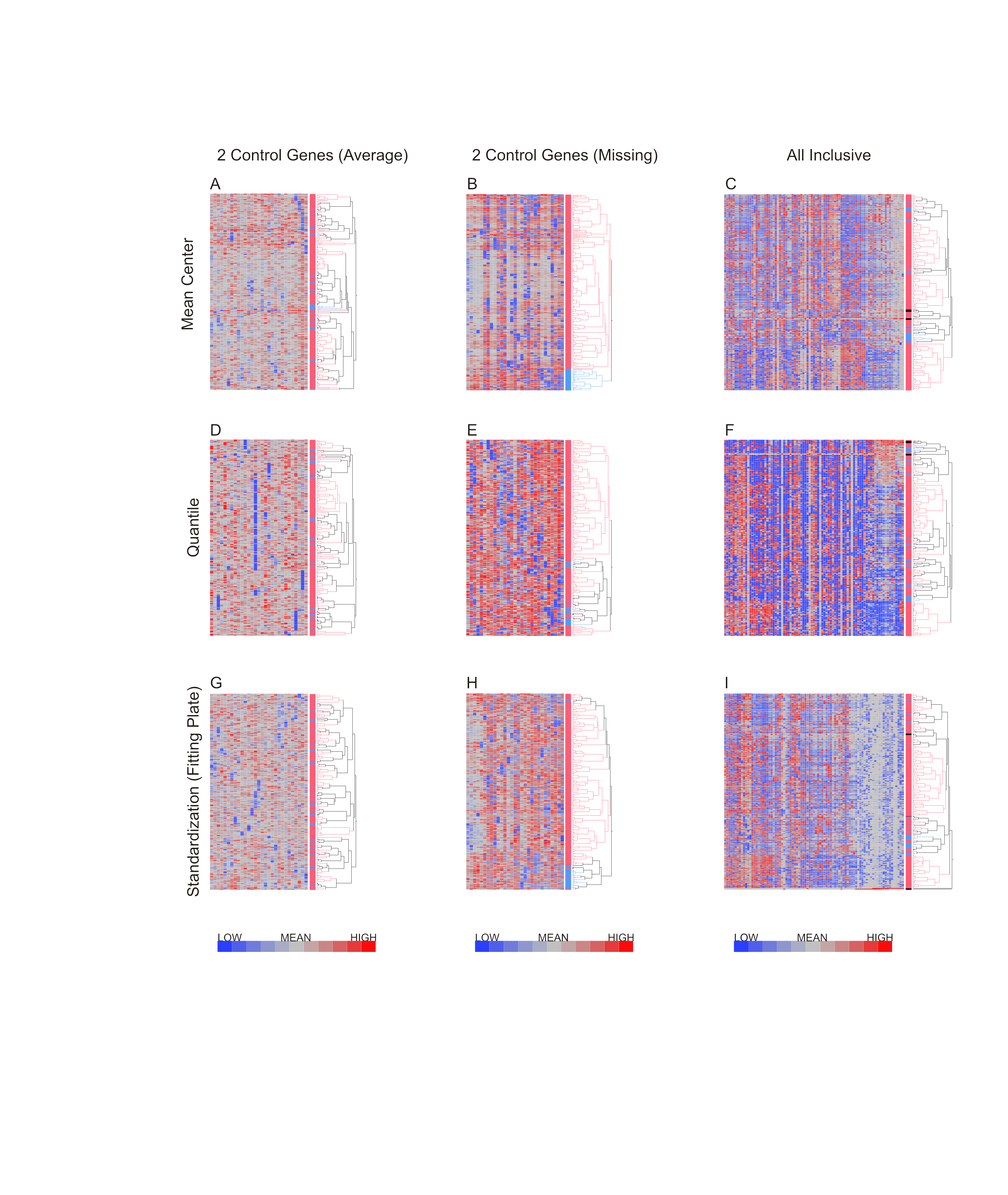

Supplement: Figure S1 — The primary data analysis method chosen affects the subsequent results. [file peerj-02-452-s001.png]
