# Supplementary material for: Single cell transcriptional analysis reveals novel innate immune cell types
Source: PeerJ. 2014 Jun 24;2:e452. doi: 10.7717/peerj.452 (PMC4081288; doi:10.7717/peerj.452)
Supplement: Table S2 — All gene targets included in the neutrophil panel and the T lymphocyte panel for qRT-PCR array. [file peerj-02-452-s002.docx]

Supplementary Table S2 - Gene Targets

| A Neutrophil Gene Targets | | | |
| --- | --- | --- | --- |
|  | |  | |
| **Gene name** | | **Gene Product** | |
| AKT1 | | V-akt murine thymoma viral oncogene homolog 1 (AKT1) | |
| AQP9 | | Aquaporin 9 | |
| B2M | | MHCI beta microglobulin | |
| BCLAF1 | | BCL2-associated transcription factor 1 | |
| BIN1 | | Bridging integrator 1 | |
| BTK | | Bruton agammaglobulinemia tyrosine kinase | |
| C5AR1 | | Complement component 5a receptor 1 | |
| CASP8 | | Caspase 8 | |
| CASP9 | | Caspase 9 | |
| CD14 | | CD14 | |
| CD4 | | CD4 | |
| CD46 | | CD46 | |
| CD83 | | CD83 | |
| CDC42 | | CDC42 | |
| CSF2RA | | CD116 | |
| CTNNB1 | | Beta-catenin | |
| DDX58 | | DEAD (Asp-Glu-Ala-Asp) box polypeptide 58 | |
| DICER1 | | Dicer 1, ribonuclease type III | |
| DYRK1A | | Dual-specificity tyrosine-(Y)-phosphorylation regulated kinase 1A | |
| EIF2AK2 | | Eukaryotic translation initiation factor 2-alpha kinase, PKR | |
| EIF4B | | Eukaryotic translation initiation factor 4B | |
| EIF4G1 | | Eukaryotic translation initiation factor 4 gamma 1 | |
| ELK1 | | ETS domain-containing protein Elk-1 | |
| FBXL5 | | F-box and leucine-rich repeat protein 5 | |
| FCGR1A | | CD64 | |
| FCGR2A | | CD32 | |
| FCGR3B | | CD16b | |
| FOS | | c-Fos (FBJ murine osteosarcoma viral oncogene homolog) | |
| GRB2 | | Growth factor receptor-bound protein 2 | |
| GSK3B | | Glycogen synthase kinase 3 beta | |
| HERC5 | | Hect domain and RLD 5 | |
| HERPUD2 | | Homocysteine-responsive endoplasmic reticulum-resident ubiquitin-like domain member 2 protein | |
| HIF1A | | Hypoxia-inducible factor 1-alpha | |
| HLA-A | | MHCI alpha chain | |
| HLA-DRA | | HLA class II histocompatibility antigen, DR alpha chain | |
| HNRPK | | Heterogeneous nuclear ribonucleoprotein K | |
| HSD17B11 | | Hydroxysteroid (17-beta) dehydrogenase 11 | |
| HSPB1 | | Heat shock protein beta-1, Heat shock protein 27 (Hsp27) | |
| IFIT1B | | Interferon-induced protein with tetratricopeptide repeats 1B | |
| IFIT2 | | Interferon-induced protein with tetratricopeptide repeats 2 | |
| IFNGR1 | | Interferon gamma receptor 1 | |
| IL17RD | | Interleukin 17 receptor D | |
| IL1B | | Interleukin 1, beta | |
| IL2RB | | Interleukin 2 receptor, beta | |
| IL6R | | IL-6 receptor | |
| IL8 | | IL-8 (CXCL8) | |
| IL8RA | | IL-8 receptor, alpha (CXCR1) | |
| ILK | | Integrin-linked kinase | |
| IMP3 | | U3 small nucleolar ribonucleoprotein protein IMP3 | |
| IRAK1 | | Interleukin-1 receptor-associated kinase 1 | |
| IRF1 | | Interferon regulatory factor 1 | |
| CD44 | | CD44 | |
| ITGAL | | CD11a (Integrin alpha L chain0 | |
| ITGAM | | Integrin Alpha-M (CD11b) | |
| ITGB2 | | CD18 ( Integrin, beta 2) | |
| MAPK3 | | Mitogen-activated protein kinase 3 | |
| MBOAT7 | | Membrane bound O-acyltransferase domain containing 7 | |
| MCL1 | | Myeloid cell leukemia sequence 1 (BCL2-related) | |
| MYD88 | | Myeloid differentiation primary response gene (88) | |
| NDEL1 | | NudE nuclear distribution gene E homolog (A. nidulans)-like 1 | |
| NFKB1 | | NF Kappa B | |
| NUMB | | NUMB homolog | |
| OASL | | 2'-5'-oligoadenylate synthetase-like | |
| OCIAD1 | | OCIA domain containing 1 | |
| OR2W3 | | Olfactory receptor, family 2, subfamily W, member 3 | |
| PAK1 | | Serine/threonine-protein kinase PAK 1 | |
| PDPK1 | | 3-phosphoinositide dependent protein kinase-1 | |
| PHC2 | | Polyhomeotic homolog 2 | |
| CREB | | cAMP response element-binding protein | |
| PIK3CG | | Phosphatidylinositol-4,5-bisphosphate 3-kinase catalytic subunit gamma isoform | |
| PTEN | | Phosphatase and tensin homolog | |
| PTPN11 | | Tyrosine-protein phosphatase non-receptor type 11 | |
| RAC1 | | Ras-related C3 botulinum toxin substrate 1 | |
| RHEB | | GTP-binding protein Rheb (Ras homolog enriched in brain) | |
| RHOA | | Ras homolog gene family, member A | |
| SAE1 | | SUMO1 activating enzyme subunit 1 | |
| SAMD9L | | Sterile alpha motif domain containing 9-like | |
| SELENBP1 | | Selenium binding protein 1 | |
| SELL | | CD62 ligand | |
| SERINC3 | | Serine incorporator 3 | |
| SHC1 | | SHC-transforming protein 1 | |
| SP3 | | Sp3 transcription factor | |
| SRF | | Srf serum response factor | |
| STK17B | | Serine/threonine kinase 17b | |
| STX3 | | Syntaxin 3 | |
| STXBP3 | | Syntaxin binding protein 3 | |
| TLR4 | | Toll-like receptor 4 | |
| TLR6 | | Toll-like receptor 6 | |
| TLR8 | | Toll-like receptor 8 | |
| TRAF6 | | TNF receptor-associated factor 6 | |
| TRIM33 | | Tripartite motif containing 33 | |
| TSC1 | | Tuberous sclerosis protein 1 | |
| TSEN34 | | tRNA splicing endonuclease 34 homolog | |
| WASPIP | | WAS/WASL interacting protein family, member 1 | |
| ZDHHC18 | | Zinc finger, DHHC-type containing 18 | |
| ZFAND5 | | Zinc finger, AN1-type domain 5 | |
|  | | |  |
|  |  | |  |
| B T Lymphocyte Gene Targets | | |  |
|  |  | |  |
| **Gene name** | **Gene Product** | |  |
| AKT3 | Akt3 | |  |
| ATP2B1 | PMCA | |  |
| B3GAT1 | CD57 | |  |
| [BCL2](http://www.proteinatlas.org/ENSG00000171791) | Bcl-2 | |  |
| CAMK4 | Calcium/calmodulin-dependent protein kinase type IV | |  |
| CCR5 | C-C chemokine receptor type 5 | |  |
| CCR7 | C-C chemokine receptor type 7 | |  |
| CD27 | CD27 | |  |
| CD28 | CD28 | |  |
| CD3D | CD3 delta | |  |
| CD4 | CD4 | |  |
| CD40LG | CD40 ligand | |  |
| CD8B | CD8, beta | |  |
| CDKN2A | Cyclin-dependent kinase inhibitor 2A, p16ink4A | |  |
| CREB1 | CAMP responsive element binding protein 1 | |  |
| CTLA4 | CD152 | |  |
| CXCR3 | Chemokine (C-X-C motif) receptor 3 | |  |
| CXCR4 | Chemokine (C-X-C motif) receptor 4, CXCR4 | |  |
| CXCR6 | Chemokine (C-X-C motif) receptor 6 | |  |
| CXCR7 | Chemokine (C-X-C motif) receptor 7 | |  |
| DUOX1 | Dual oxidase 1 | |  |
| DUSP2 | Dual specificity protein phosphatase 2 | |  |
| DUSP3 | Dual specificity protein phosphatase 3 | |  |
| FAS | CD95 | |  |
| FASLG | Fas ligand | |  |
| FCAR | CD89 | |  |
| FYN | Proto-oncogene tyrosine-protein kinase Fyn | |  |
| GLRX2 | Glutaredoxin 2 | |  |
| GZMB | Granzyme B | |  |
| HIF1A | Hypoxia-inducible factor 1-alpha, HIF-1alpha | |  |
| IFNGR1 | Interferon gamma receptor, IFN gamma receptor | |  |
| IL10 | Interleukin-10, IL-10 | |  |
| IL10RA | Interleukin-10 receptor, alpha, CDw210 | |  |
| IL10RB | Interleukin-10 receptor, beta | |  |
| IL12RB2 | Interleukin 12 receptor, IL-12 receptor | |  |
| IL17A | Interleukin 17, IL-17 | |  |
| IL18R1 | Interleukin 18 receptor 1, IL-18 receptor 1 | |  |
| [IL1R1](http://www.proteinatlas.org/ENSG00000115594) | CD121a (IL-1RI and IL-1RII) | |  |
| IL2 | Interleukin-2, IL-2 | |  |
| IL21R | Interleukin-21 receptor, IL-21 receptor | |  |
| IL2RA | CD25 | |  |
| IL2RB | Interleukin-2 receptor, beta, Interleukin-15 receptor | |  |
| IL2RG | Interleukin-2 receptor, gamma, CD132 | |  |
| IL4R | Interleukin-4, IL-4 | |  |
| IL6 | Interleukin 6, IL-6 | |  |
| IL7R | Interleukin-7 receptor, alpha, IL-7 receptor | |  |
| ITK | IL2-inducible T-cell kinase | |  |
| JAK1 | Janus kinase 1 | |  |
| JAK3 | Janus kinase 3 | |  |
| KLRC1 | Killer cell lectin-like receptor subfamily C, member 1 | |  |
| LAG3 | CD223 | |  |
| LAT | Linker for Activation of T cells, LAT | |  |
| LCK | Lymphocyte-specific protein tyrosine kinase | |  |
| LTA, TNFB | Tumor Necrosis Factor beta, TNF beta | |  |
| MAP3K5 | Apoptosis signal-regulating kinase 1 | |  |
| MAPK1 | Mitogen-activated protein kinase 1, ERK2 | |  |
| MAPK9 | Mitogen-activated protein kinase 9, JNK2 | |  |
| MPO | Myeloperoxidase | |  |
| [NFE2L2](http://www.proteinatlas.org/ENSG00000116044) | Nuclear factor (erythroid-derived 2)-like 2 | |  |
| NPAT | CD245 | |  |
| NRAS | N-Ras | |  |
| ORAI1 | Calcium release-activated calcium channel protein 1 | |  |
| PIK3CD | PI3K, catalytic, delta polypeptide | |  |
| PLCG1 | PLC gamma | |  |
| Prf1 | Perforin 1 | |  |
| PRKCQ | PKC theta | |  |
| PTEN | Phosphatase and tensin homolog, PTEN | |  |
| PTPN1 | Protein-tyrosine phosphatase 1B, PTP1B | |  |
| PTPN11 | Tyrosine-protein phosphatase non-receptor type 11, SHP-2 | |  |
| PTPN22 | Protein tyrosine phosphatase, non-receptor type 22, LYP | |  |
| PTPN6 | Src homology region 2 domain-containing phosphatase-1, SHP1 | |  |
| RAC2 | Ras-related C3 botulinum toxin substrate 2, Rac2 | |  |
| RPP38 | p38 | |  |
| SH2D1A | SH2 domain-containing protein 1A, sphingolipid activator protein-1, SAP | |  |
| SOD2 | Superoxide dismutase , SOD2 | |  |
| SOS2 | SOS-2 | |  |
| STAT3 | Signal transducer and activator of transcription 3, STAT3 | |  |
| STAT5a | Signal transducer and activator of transcription 5A, STAT5a | |  |
| STAT5b | Signal transducer and activator of transcription 5B, STAT5b | |  |
| STAT6 | Signal transducer and activator of transcription 6, STAT6 | |  |
| STIM1 | Stromal interaction molecule , STIM1 | |  |
| TCRG | Tcell Receptor, gamma, TCR gamma | |  |
| TERF1 | Telomeric repeat-binding factor 1, TERF1 | |  |
| TGFB1 | TGF beta 1 | |  |
| TGFBR2 | TGF beta receptor 2 | |  |
| TLR4 | Toll-like receptor 4, TLR4 | |  |
| TLR5 | Toll-like receptor 5, TLR5 | |  |
| TLR7 | Toll-like receptor 7,TLR7 | |  |
| TNF, TNFA | Tumor Necrosis Factor alpha, TNF alpha | |  |
| TNFRSF8 | CD30 | |  |
| [TNFRSF9](http://www.proteinatlas.org/ENSG00000049249) | 4-1BB, CD137 | |  |
| TRAF6 | TNF receptor associated factor 6, TRAF6 | |  |
| TXN2 | Thioredoxin 2, Trx2 | |  |
| VAV1 | VAV1 | |  |
| WNT3 | WNT3 | |  |
| ZAP70 | Zeta-chain-associated protein kinase 70, Zap70 | |  |
