# Supplementary material for: Single cell transcriptional analysis reveals novel innate immune cell types
Source: PeerJ. 2014 Jun 24;2:e452. doi: 10.7717/peerj.452 (PMC4081288; doi:10.7717/peerj.452)
Supplement: Table S3 [file peerj-02-452-s003.docx]

| Supplementary Table 1 - Number of Cells and Genes in Data Sets | | | |
| --- | --- | --- | --- |
|  |  |  |  |
|  | Neutrophils |  | T Lymphocytes |
| Raw Data |  | Raw Data |  |
| Cells | 220 | Cells | 251 |
| Genes | 93 | Genes | 94 |
| Supervised |  | 2 Control Genes: Average |  |
| Cells | 202 | Cells | 244 |
| Genes | 59 | Genes | 38 |
| 2 Control Genes * |  | 2 Control Genes*: Missing |  |
| Cells | 208 | Cells | 244 |
| Genes | 62 | Genes | 38 |
| All Inclusive |  | All Inclusive |  |
| Cells | 220 | Cells | 247 |
| Genes | 81 | Genes | 94 |
